# The Phrenic Component of Acute Schizophrenia – A Name and Its Physiological Reality

**DOI:** 10.1371/journal.pone.0033459

**Published:** 2012-03-16

**Authors:** Karl-Jürgen Bär, Tobias Rachow, Steffen Schulz, Katharina Bassarab, Stefanie Haufe, Sandy Berger, Kathrin Koch, Andreas Voss

**Affiliations:** 1 Pain and Autonomic Integrative Research (PAIR), Department of Psychiatry and Psychotherapy, University Hospital, Jena, Germany; 2 Department of Medical Engineering and Biotechnology, University of Applied Sciences, Jena, Germany; 3 Department of Psychiatry and Psychotherapy, University Hospital, Jena, Germany; Baylor College of Medicine, United States of America

## Abstract

Decreased heart rate variability (HRV) was shown for unmedicated patients with schizophrenia and their first-degree relatives, implying genetic associations. This is known to be an important risk factor for increased cardiac mortality in other diseases. The interaction of cardio-respiratory function and respiratory physiology has never been investigated in the disease although it might be closely related to the pattern of autonomic dysfunction. We hypothesized that increased breathing rates and reduced cardio-respiratory coupling in patients with acute schizophrenia would be associated with low vagal function. We assessed variability of breathing rates and depth, HRV and cardio-respiratory coupling in patients, their first-degree relatives and controls at rest. Control subjects were investigated a second time by means of a stress task to identify stress-related changes of cardio-respiratory function. A total of 73 subjects were investigated, consisting of 23 unmedicated patients, 20 healthy, first-degree relatives and 30 control subjects matched for age, gender, smoking and physical fitness. The LifeShirt®, a multi-function ambulatory device, was used for data recording (30 minutes). Patients breathe significantly faster (p<.001) and shallower (p<.001) than controls most pronouncedly during exhalation. Patients' breathing is characterized by a significantly increased amount of middle- (p<.001), high- (p<.001), and very high frequency fluctuations (p<.001). These measures correlated positively with positive symptoms as assessed by the PANSS scale (e.g., middle frequency: r = 521; p<.01). Cardio-respiratory coupling was reduced in patients only, while HRV was decreased in patients and healthy relatives in comparison to controls. Respiratory alterations might reflect arousal in acutely ill patients, which is supported by comparable physiological changes in healthy subjects during stress. Future research needs to further investigate these findings with respect to their physiological consequences for patients. These results are invaluable for researchers studying changes of biological signals prone to the influence of breathing rate and rhythm (e.g., functional imaging).

## Introduction

Somatic consequences of chronic mental diseases have been brought into focus during the last years. In patients suffering from schizophrenia, a high incidence of metabolic syndrome or coronary heart disease (CHD) have frequently been reported [1], [2]. However, the question as to whether long-term consequences of the disease - such as a lack of daily physical activity, decreased access to medical services or chronic side effects of treatment - were causing these conditions, or whether these co-morbidities indicate etiologic overlaps, is difficult to resolve [3]. A growing body of evidence has accumulated describing decreased heart rate variability (HRV) being associated with positive and negative symptoms in young patients with schizophrenia and - more intriguingly - in their healthy relatives [4], [5], [6]. While a high degree of HRV indicates healthy cardiac regulation (including a balanced influence of parasympathetic and sympathetic modulation on the heart) decreased HRV is associated with a high incidence of cardiac morbidity and mortality and may predispose patients to cardiac events such as ventricular fibrillation [3], [7]. In contrast to autonomic function in primary medical conditions, it has been suggested that decreased HRV in patients with schizophrenia is caused by a decreased interaction between prefrontal brain areas and subcortical structures, leading to arousal and an autonomic imbalance characterized by vagal withdrawal and sympathetic predominance [8], [9]. This autonomic pattern was demonstrated in unmedicated patients with schizophrenia by examining the heart, the pupil, the stomach or the cadio-respiratory system [6], [10], [11]. However, another important player, namely the respiratory function, which is a key regulatory centre for body homeostasis [12], has very rarely been assessed in the disease, although it might point to the central origin of the autonomic disturbance and might serve as an entrance to influence autonomic function directly by means of biofeedback interventions.

Breathing movements are facilitated by a pontine–medullary respiratory network generating rhythmic patterns of in- and exhalation [13]. This network originates within the interconnected bilateral columns of the medullary neurons as well as in the ventral respiratory columns (VRCs) and is controlled by inputs from other medullary structures, including the retrotrapezoid nucleus (RTN), raphe nuclei and other rostral pontine circuits. Breathing is not only controlled by metabolic demands, but responds to changes in emotions, such as sadness, anxiety and fear [12]. This relation between breathing and emotion can also be seen in the close connection between the neurons in the amygdala complex and the respiratory regions in the medulla and pons [14], [15]. Given the close interrelationship between emotion and breathing, it is intriguing that relatively few studies have investigated alterations in breathing rates and rhythms in patients with schizophrenia [16], [17], [18]. Those early reports that do exist suggested that increased breathing rates are accompanied by reduced variability [16], [17]. This was corroborated by a recent study showing the close interrelation between positive symptoms and non-linear respiratory patterns [18].

Our study set out to analyze and describe in detail the respiratory function and its corresponding cardiac regulation in patients suffering from acute schizophrenia, their healthy first-degree relatives and controls. We hypothesized that we would find increased breathing rates in patients as an indication of high internal arousal, accompanied by decreased cardio-respiratory coupling. Therefore, we correlated psychopathology with parameters of respiration, and included an additional stress experiment to investigate the regulation of respiration and cardio-respiratory coupling under these conditions in healthy controls. In addition, we hypothesized that increased breathing rates in patients would not explain the reduced HRV that was previously described for patients and healthy first-degree relatives.

## Materials and Methods

### First experiment (patients, relatives and controls)

We included 23 patients (mean age 30.4 years; 19–58 years) suffering from paranoid schizophrenia, 20 of their healthy first-degree relatives (6 siblings, 14 offspring; mean age 31.6 years; 19–56 years) and 30 healthy controls (mean age 29.9 years; 22–58 years) matched to relatives with respect to age, sex, weight, smoking habits and education (see Table 1). Patients were included only when they had not taken any medication for at least 8 weeks. 8 patients were investigated during the first episode and were followed-up for 6 months. Serum drug levels were controlled for legal drugs (e.g., antipsychotics, antidepressants, benzodiazepines) and illegal substances (e.g. cannabis). In accordance with our inclusion criteria, only subjects with negative results were included in the study. A clinical ECG was recorded prior to the investigation and evaluated by a cardiologist. Similarly, patients were clinically assessed for the presence of any lung disease. Diagnosis of paranoid schizophrenia was established when patients fulfilled DSM-IV criteria (Diagnostic and statistical manual of mental disorders, 4^th^ edition, [19]). The semi-structured clinical interview SCID-1 was used for patients to approve the clinical diagnosis [19]. Psychotic symptoms were quantified using the Positive and Negative Syndrome Scale (PANSS, [20]).

**Table 1:** Clinical and demographic data of participants.

*Parameter*	*Controls*	*Relatives*	*Patients*
Number of participants	n = 30	n = 20	n = 23
Male/Female	16/14	12/8	13/10
Age [in years] mean ± SD (min-max)	29.9±9.5(22–58)	31.6±10.7(19–56)	30.4±10.3(19–58)
Body Mass Index, mean (SD)	23.6±3.9	25.6±4.7	23.9±4.4
**Education**			
8–10 years at school, No.	n = 2	n = 1	n = 5
12 years at school (A-Level), No.	n = 28	n = 19	n = 18
Attended university, No.	n = 23	n = 10	n = 5
Smoker/Non-Smoker	17/13	4/16	16/7
<5 cigarettes/day, No.	n = 1	n = 1	n = 1
5–10 cigarettes/day, No.	n = 4	n = 1	n = 1
>10 cigarettes/day, No.	n = 13	n = 2	n = 14
**Coffee Consumption**			
No coffee consumption, No.	n = 5	n = 6	n = 3
1 cup/day, No.	n = 5	n = 5	n = 3
2 cups/day, No.	n = 15	n = 5	n = 9
≥3 cups/day, No.	n = 5	n = 4	n = 8
**Alcohol Consumption**			
Alcohol consumption (g/day)	11.9±12.5	13.1±15.4	19.1±18.1
**Sport**			
No sport, No.	n = 6	n = 12	n = 12
<2 h/week, No.	n = 9	n = 2	n = 4
2–5 h/week, No.	n = 10	n = 3	n = 5
>5 h/week, No.	n = 5	n = 3	n = 2
PANSS, mean (min-max)	n.a.	n.a.	85.7 (43–124)
SANS, mean (min-max)	n.a.	n.a.	49.6 (14–81)
SAPS, mean (min-max)	n.a.	n.a.	60.9 (6–108)

PANSS - Positive and negative syndrome; scale; SANS - Scale for the assessment of negative symptoms; SAPS - Scale for the assessment of positive symptoms; n.a. – not applicable.

Control subjects were recruited from hospital staff (n = 4), medical students (n = 6) and the local community (n = 20). A careful interview and a clinical investigation were performed for all relatives and controls to exclude any potential psychiatric or other diseases as well as any interfering medications. The Structured Clinical Interview SCID II and a personality inventory (Freiburger Persönlichkeitsinventar, FPI) were additionally applied for relatives and controls to detect personality traits or disorders which might influence autonomic function [21]. This study complied with the Declaration of Helsinki. All participants gave written informed consent to a protocol approved by the Ethics Committee of the University Hospital, Jena. Patients and relatives were advised that the refusal of participating in this study would not affect future treatment. Patients were only included after a psychiatrist (S.B.) certified their ability to give full consent to the study protocol. Patients unable to provide informed consent were not included.

### Data Acquisition and Pre-Processing

Investigations were performed between 3 and 6 p.m. in a quiet room which was kept comfortably warm (22–24°C) and began after subjects were connected to the equipment and had rested in supine position for 10 minutes to allow for adjustment. Subjects were asked to relax and to breathe normally to avoid hyperventilation. No further instruction for breathing was given.

A high resolution electrocardiogramm was recorded for 30 minutes as previously shown [22]. The LifeShirt® (Vivometrics, Inc., Ventura, CA, U.S.A.), a multi-function ambulatory device and consisting of a Lycra® garment, data recorder and computer-based analysis software (VivoLogic) were used [23], [24]. Respiratory inductive plethysmography, the core system which has been demonstrated to provide an accurate non-invasive assessment of respiratory patterns, was employed [25]. In order to obtain the ventilated volume for further analysis, the device was calibrated in two different successive steps as recommended by the manufacturer. Initially, a qualitative diagnostic calibration procedure had to be performed. Therefore, a period of about 5 minutes of calm breathing was used to compute a calibration factor [26]. Additionally, a second procedure of calibration which is based on defined breaths of a fixed volume was conducted [27]. Here, all subjects had to perform a set of 7 breaths of a defined tidal volume of 800 ml by breathing it in a plastic bag while wearing a nose clip, first in a supine and then in a sitting position. The basic concept of this calibration is a two-compartment model of the respiratory system consisting of thoracic and abdominal compartments. In addition, the saturation of blood oxygenation (SpO_2_) was assessed by means of pulse oxymetry.

Heart rate time series consisting of successive beat-to-beat intervals (RRI) were extracted from the raw data records. The respiration phase was calculated to generate the interval times between consecutive breathing cycles for further analysis. Afterwards, these time series were filtered by applying an adaptive variance estimation algorithm to remove and interpolate ventricular premature beats and artifacts (e.g. movement, electrode noise and extraordinary peaks).

### Respiratory parameters

Raw respiratory data were processed to obtain respiratory parameters. Both the breathing rate and volume were analyzed. The breathing rate was defined as the number of breaths per minute (Br/min). In addition, inhalation and exhalation time intervals were determined for each breath in order to calculate the ratio of inhalation to exhalation time. According to HRV parameters, the variability of the respiration rate was computed by calculating the time domain parameter RMSSD_Resp_ (root mean square of successive differences) as well as the following frequency domain parameters: low frequency (LF_Resp_, 0–0.1 Hz), mid frequency (MF_Resp_, 0.1–0.25 Hz), high frequency (HF_Resp_ 0.25–0.4 Hz) and very high frequency (VHF_Resp_, 0.4–.06 Hz). In addition, the minute ventilation (MV) and the rapid shallow breathing index were computed. This is defined as the quotient of the breathing rate and the tidal volume of each breath, as described previously [28]. Variability parameters of tidal volume (TV) were calculated using a similar method to the variability of respiration rate (RMSSD_TV_, LF_TV_, HF_TV_).

### Parameters of time and frequency domain of heart rate variability

We obtained the basic heart rate (HR), and the RMSSD_HRV_ (root mean of squared successive difference) as a time domain parameter of heart rate variability, as well as low frequency (LF_HRV_, 0.04–0.15 Hz) and high frequency parameters (HF_HRV_, 0.15–0.40 Hz) of the frequency domain [29]. In addition, respiratory sinus arrhythmia (RSA) and compression entropy (HC_HRV_) were calculated.

### Respiratory sinus arrhythmia (RSA)

The RSA was quantified as a measure of cardiac vagal tone, which is a cardio-respiratory phenomenon characterized by HR (or R-R interval (RRI)) fluctuations that are in phase with inhalation and exhalation [30]. The Life vest® uses the peak-to-valley method for assessing each breathing cycle [31]. In order to account for the strong dependency of the RSA on breathing rates, a transfer curve was calculated for each group, illustrating single breathing rates and corresponding RSA values [32], [33]. We performed an averaging for every breathing rate which finally allowed us to plot the transfer curves as a regression curve.

### Compression entropy (HC_HRV_)

Besides linear computational algorithms, nonlinear methods were applied. Lempel and Ziv (1977) introduced a universal algorithm (ZIP) for lossless data compression using string matching on a sliding window. With some modifications, this algorithm can be applied for the analysis of heart beat time series [34], [35]. Here, the Hc quantifies the extent to which data from heart beat time series can be compressed, i.e., how often repetitive sequences occur (Hc is calculated as the ratio of the length of the compressed data series divided by the length of the original data series). Reduced short-term fluctuations (reduced variability) of HR result in increased compression. Entropy reduction appears to reflect a change in sympathetic/parasympathetic HR control [36].

### Interaction between heart rate and respiration - Joint Symbolic Dynamics

For a nonlinear interaction analysis between blood pressure and heart rate time series, the method of Joint Symbolic Dynamics (JSD) was introduced, which is based on the analysis of bivariate dynamic processes by means of symbols [37].

JSD considers short-term beat-to-beat changes, allowing the assessment of overall cardio-respiratory short-term coupling.

The usefulness of JSD for analyzing cardio-respiratory interdependencies from heart rate interbeat intervals and breath rate time series was successfully demonstrated in some recent studies [38], [39].

The time series of heart rate interbeat intervals (HR) and breath rates (RESP) (*x^HR^*, *x^RESP^*) were transformed into symbol sequences (*s^HR^*, *s^RESP^*) with a given alphabet A = [0.1] using the symbol ‘1’ for increasing values and symbol ‘0’ for decreasing and unchanged values. Thus, short patterns (words *w*) of symbol sequences with a word-length of three symbols were formed. From all single word types, the normalized joint probability (*p(w_i,j_)*) of occurrence was estimated using an 8×8 word distribution density matrix *W* (columns – HR, rows – RESP) ranges from [000, 000]^T^ to [111, 111]^T^. Furthermore, the Shannon entropy (*JSD_shannon_*) within this word distribution density matrix was calculated as a measure of the overall complexity of the cardio-respiratory couplings:
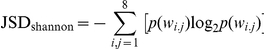



### Interaction between heart rate and respiration - Cross Conditional Entropy

The Cross Conditional Entropy (CCE) provides a quantification of the degree of coupling between two signals [40]. Synchronization occurs when interactive dynamics between two signals are repetitive. Initially, the signals are embedded in multiple dimensions. For each dimension, the conditional entropy (CE), modified from the Shannon entropy, is calculated as a measure of complexity. CE is a process of sorting and counting mixed patterns and represents the amount of information carried by the present sample of signal *x* when the pattern *L-1* from signal *y* is known. Based on CE, one can define the uncoupling function (UF) as a measure quantifying the amount of information exchanged between the heart rate interbeat intervals and the breath rate time series. The larger the uncoupling functions are, the more uncoupled are the two signals. UF is 1 when the heart rate interbeat intervals and the breath rate time series are independent, while it is 0 for completely synchronized periodic dynamics [41].

### Second experiment (stress experiment)

Control subjects underwent a second investigation: the previously published Mannheim Multicomponent Stress Test (MMST) [42]. This test exploits a combination of mental stress, noise and emotional pictures presented in the background. After connecting participants to the equipment, a resting period of 15 minutes was allowed, followed by a 10-minute baseline measurement. The induction of stress was given for 10 minutes followed by a 10-minute resting phase. Exactly the same parameters were calculated for patients, healthy relatives and controls as in experiment 1 and were calculated as described above. For clarity, only a subset of parameters will be presented.

### Statistical analyses

For statistical analysis, SPSS for Windows (version 17.0) was used. All parameters were tested for normal distribution by Kolmogorov-Smirnov tests.

### First experiment

Three multivariate analyses of covariance (MANCOVAs) were performed according to the physiological origin of obtained parameters respiration, HRV and the cardio-respiratory interaction respectively, using age as a covariate. All MANCOVAs were followed by analyses of covariance (ANCOVAs) for single parameters and a Bonferroni-Holm corrected pair-wise comparison. To better understand the interdependence of respiration and cardiac vagal modulation, we performed two additional MANCOVAs using either an index of vagal function or respiration rate as covariate.

**Figure 1:**
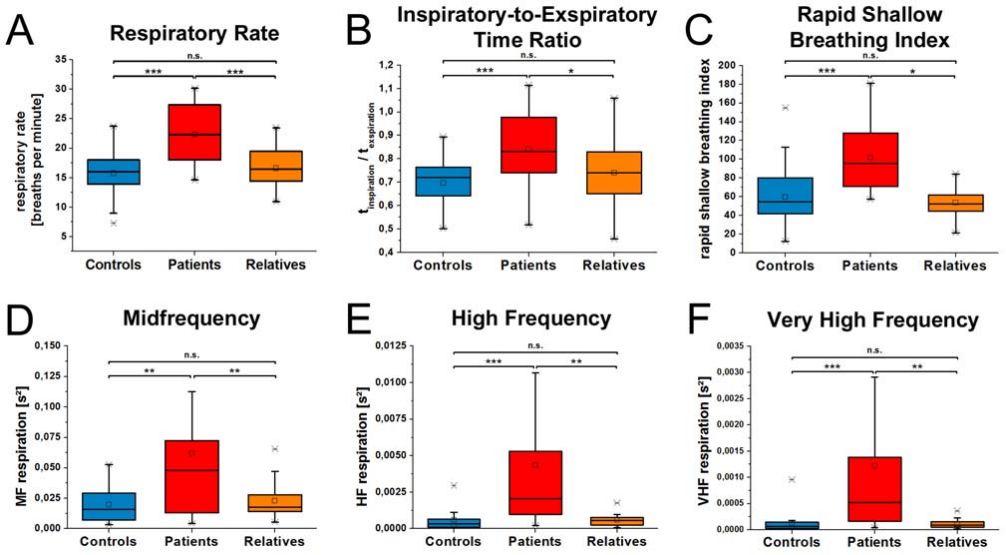
Parameters of respiratory analysis of controls, patients and relatives are presented. The pattern of significantly altered values of patients in comparison to controls is represented in A–F. The increased breathing rate of patients is presented in A. As shown in B, the inspiratory-to-expiratory time ratio is increased in patients indicating a reduction of the exhalation time. Patients breathe shallowly as indicated in C. Most pronounced are increased frequency bands of respiration as indicated in D–F. Boxes indicate data between the 25th and 75th percentile with the horizontal bar reflecting the median (□ = mean; - = 1st and 99th percentile; x = minimum and maximum of data). Significant differences of Bonferoni corrected pair-wise comparisons are indicated: * p<.05; ** p<.01; *** p<.001.

The first MANCOVA was applied including the factor GROUP (controls, patients, relatives) for parameters of respiration (including breathing rate and variability parameters: RMMSD_Resp_, LF_Resp_, MF_Resp_, HF_Resp_, VHF_Resp_, rapid shallow breathing index, ratio of inspiration and exhalation, minute volume and tidal volume (TV) and its variability parameters: RMMSD_TV_, LF_TV_, HF_TV_). Similarly, analyses of covariance (ANCOVAs) were calculated for single parameters of relatives, controls, and patients. To reveal differences in single parameters between all groups included, a Bonferroni-Holm corrected pair-wise comparison was performed as a post hoc test. Results are given in figure 1 and table 2.

**Table 2:** Obtained parameters of experiment 1 and 2 (not included in figures).

*Experiment 1*	Controls (C)	Patients (P)	Relatives (R)	p-value		
				P vs. C	R vs. C	P vs. R
RMSSD_Resp_ [s]	0.20±0.065	0.254±0.138	0.186±0.110	n.s.	n.s.	n.s.
LF_Resp_ [s^2^]	1.12±2.35	1.14±2.08	1.01±2.35	n.s.	n.s.	n.s.
Tidal Vol [ml]	345±165	235±87	371±126	<.003	n.s.	<.001
Minute Vent [l]	5.33±2.05	6.17±3.15	6.11±2.20	n.s.	n.s.	n.s.
RMSSD_TV_ [ml]	0.33±0.29	0.238±0.189	0.323±0.301	n.s.	n.s.	n.s.
LF_TV_ [ml^2^]	0.02±0.018	0.040±0.048	0.019±0.032	n.s.	n.s.	n.s.
HF_TV_ [ml^2^]	4 10^−4^±3 10^−4^	7 10^−4^±9 10^−4^	4 10^−4^±6 10^−4^	n.s.	n.s.	n.s.
LF_HRV_ [ms^2^]	254±180	131±109	120±91	<.005	<.005	n.s.
SpO_2_ [%]	95.1±2.7	94.6±2.1	94.0±2.3	n.s.	n.s.	n.s.

*Experiment 2*	Baseline (B)	Stress induction (S)	Post stress (PS)	p-value	
				B vs. S	S vs. PS
Ratio I/E	0.71±0.23	0.85±0.53	0.73±0.32	<.05	n.s.
LF_Resp_ [s^2^]	1.15±0.76	0.15±0.07	0.83±0.22	n.d.	n.d.
MF_Resp_ [s^2^]	0.022±0.007	0.014±0.006	0.045±0.014	n.s.	n.s.
HF_Resp_ [s^2^]	0.002±0.001	0.001±0.0007	0.003±0.001	n.s.	n.s.
RMSSD_HRV_ [ms]	34±3.9	26.7±5.5	36.7±4.4	n.s.	<.009
Hc_HRV_	0.68±0.017	0.64±0.2	0.70±0.017	n.s.	<.001
CCE	0.16±0.017	0.11±0.011	0.12±0.019	n.d.	n.d.

Values are displayed as mean ± standard deviation, RMSSD_Resp_ = root mean square of successive differences, Resp = respiratory rate, HRV = heart rate variability, TV = tidal volume, HF high frequency, LF = low frequency, Minute Vent = minute ventilation; Tidal Vol = tidal volume, SpO_2_ = blood oxygen saturation level, ratio I/E = inspiratory to expiratory time ratio, MF = mid frequency, Hc = compression entropy, CCE = cross conditional entropy, n.s. = not significant, n.d. = not done (ANOVA not significant).

**Figure 2:**
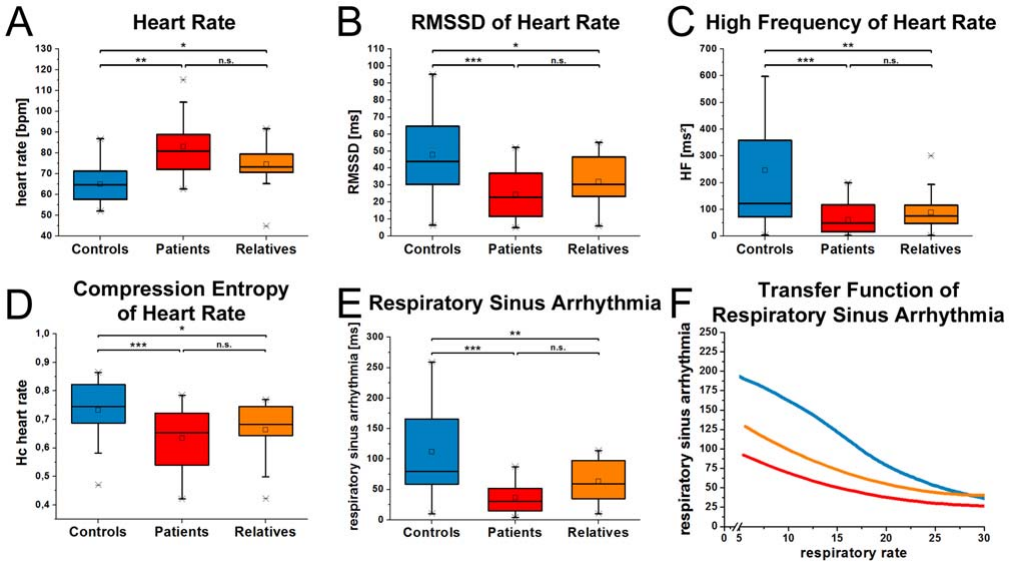
The figure indicates parameters of HRV in patients, relatives and controls. Increased mean heart rates of patients and relatives are depicted in A. Decreased parasympathetic modulation is shown in B by means of the RMSSD and in C using the high frequency band of heart rate. Reduced complexity is shown for patients and relatives in D. Respiratory sinus arrhythmia (E) indicates low cardiac vagal modulation in patients and relatives. Transfer function (F) shows that decreased cardiac vagal modulation in patients and relatives is independent from the respiratory rate. Boxes indicate data between the 25th and 75th percentile with the horizontal bar reflecting the median (□ = mean; - = 1st and 99th percentile; x = minimum and maximum of data). Significant differences of Bonferoni corrected pair-wise comparisons are indicated: * p<.05; ** p<.01; *** p<.001.

A second MANCOVA was applied using the factor GROUP (controls, patients, relatives) for parameters of HRV (mean heart rate, RMMSD_HRV_, LF_HRV_, HF_HRV_, Hc_HRV_ and RSA) of relatives, control subjects, and patients suffering from schizophrenia. Age was used as a covariate. ANCOVAs followed by Bonferroni-Holm corrected pair-wise comparisons as post-hoc analyses were performed in a similar way to the analysis of respiratory parameters. Results are indicated in figure 2 and table 2.

A third MANCOVA was computed to analyze the dynamic interaction between heart rate and breathing behavior for both the JSD Shannon and cross conditional entropy (CCE) parameters. This analysis was followed by ANCOVAs and Bonferroni-Holm corrected pair-wise comparisons for patients, relatives and control subjects.

To investigate the interrelation between cardiac vagal modulation and respiratory function on the one hand and the influence of breathing rate on HRV parameters on the other, we calculated two additional MANCOVAs. To analyze a potential effect of vagal modulation on breathing, we recalculated the MANCOVA for respiratory parameters, including RMSSD_HRV_ as a covariate. Similarly, we calculated the MANCOVA for HRV parameters using the breathing rate as a covariate.

In line with our hypothesis that internal arousal will influence respiratory measures, we correlated parameters of respiration with the subscale for positive symptoms of PANSS (P-PANSS) of patients.

The scores of the personality traits assessed in the Freiburger Persönlichkeitsinventar were compared between relatives and controls by means of a two-tailed t-test applying Bonferroni-Holm correction. These values were then correlated with autonomic parameters for the relatives and controls separately.

### Second experiment

A repeated measure multivariate analysis of variance (MANOVA) was performed to investigate an overall effect of TIME (baseline – stress task – post test phase) for all the parameters included (mean breathing rate, ratio of inspiration and exhalation, LF_Resp_, MF_Resp_, HF_Resp_, and mean heart rate, RMMSD_HRV_, RSA, Hc_HRV_ as well as JSD Shannon and CEE). This analysis was followed by ANOVAs to analyze the effect of TIME for single parameters, and Bonferroni-Holm corrected pair-wise comparisons between the baseline and the stress condition, and between stress the condition and the post-stress phase.

## Results

### First Experiment - Multivariate analysis of covariance (MANCOVA) of respiratory parameters and follow-up analysis of covariance (ANCOVA) of single parameters for all groups controlled for age

The overall MANCOVA revealed significant differences for respiratory parameters (breathing rate, rapid shallow breathing index, minute volume, ratio of inspiration and exhalation, RMMSD_Resp_, LF_Resp_, MF_Resp_, HF_Resp_, VHF1_Resp_, tidal volume, RMMSD_TV_, LF_TV_, HF_TV_) between all three groups [F(26,114) = 3.36; p<.001]. Follow-up ANCOVAs showed significant effects among all three groups for breathing rate [F(3,69) = 22.1; p<.001], rapid shallow breathing index [F(3,69) = 19.3; p<.001], ratio of inspiration and exhalation [F(3,69) = 2.8; p<.04], tidal volume [F(3,69) = 4.2; p<.02] as well as for MF_Resp_, [F(3,69) = 7.6; p<.001], HF_Resp_ [F(3,69) = 10.3; p<.001], and VHF_Resp_ [F(3,69) = 9.5; p<.001] (Figure 1A–E). No significant differences between all groups were observed for minute volume (p = .34), RMMSD_Resp_ (p = .07), LF_Resp_ (p = .97) as well as for RMMSD_TV_ (p = .5), LF_TV_ (p = .06), HF_TV_ (p = .06). Results of the Bonferoni corrected pair-wise comparisons are depicted in Figure 1 and Table 2.

### First Experiment - MANCOVA and follow-up ANCOVAs of heart rate variability for all groups controlled for age

The MANCOVA for HRV parameters showed a significant overall effect for the included HRV parameters (mean heart rate, RMMSD_HRV_, LF_HRV_, HF_HRV_, Hc_HRV_ and RSA) between groups [F(12,128) = 3.7; p<.001]. Univariate ANCOVAs for single parameters revealed highly significant differences between groups for heart rate [F(3,69) = 6.8; p<.002], for RMSSD_HRV_ [F(3,69) = 13.5; p<.001], for LF_HRV_ [F(3,69) = 7.6; p<.001], for HF_HRV_ [F(3,69) = 9.9; p<.001], for compression entropy HC_HRV_ [F(3,69) = 10.8; p<.001], and for respiratory sinus arrhythmia (RSA) [F(3,69) = 17.8; p<.001]. Figure 2 and Table 2 show results of the Bonferroni-Holm corrected pair-wise comparisons.

### First Experiment - MANCOVA and follow-up ANCOVAs of dynamic breathing interaction for all groups controlled for age

**Figure 3:**
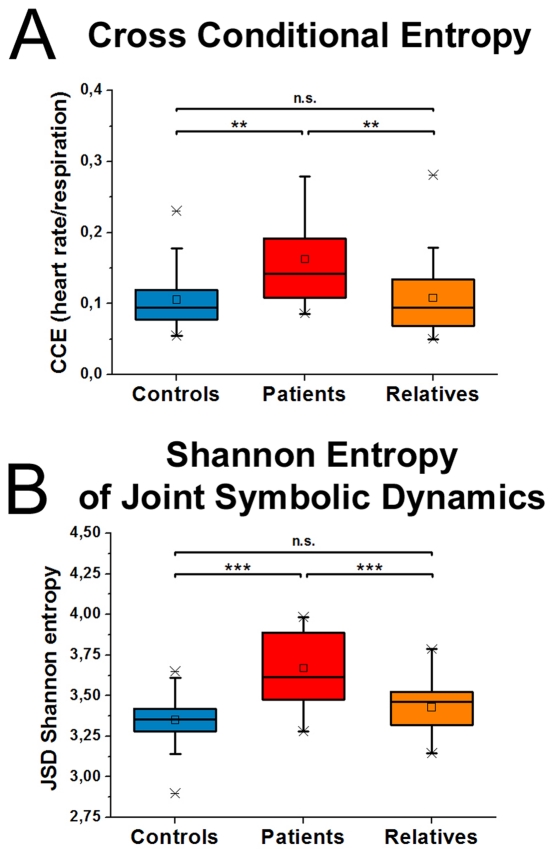
Interaction analyses of heart rate and respiration are shown. Increased uncoupling between heart rate and respiratory rate is shown in patients in comparison to controls and relatives. Both measures, cross conditional entropy (A) and shannon entropy of joint symbolic dynamics (B) provide a quantification of the degree of coupling between both signals. Boxes indicate data between the 25th and 75th percentile with the horizontal bar reflecting the median (□ = mean; - = 1st and 99th percentile; x = minimum and maximum of data). Significant differences of Bonferoni corrected pair-wise comparisons are indicated: ** p<.01; *** p<.001.

The MANCOVA for dynamic breathing interaction revealed a significant overall effect for the factor GROUP [F(4,136) = 11.7; p<.001] including both the cross-conditional entropy (CCE) and JSD Shannon parameters. Follow-up univariate ANOVAS indicated differences for both CCE [F(3,69) = 7.4; p<.001] and JSD shannon [F(3,69) = 23.6; p<.001]. Figure 3 shows results of the Bonferroni-Holm corrected pair-wise comparisons.

### First Experiment - Multivariate analysis of co-variance of parameters of respiration and dynamic interaction controlled for vagal modulation

The inclusion of RMSSD as a measure of cardiac vagal activity in the MANCOVA of respiratory parameters (see 3.1) indicated still an overall difference between groups [F(26,114) = 2.7; p<.001]. Similarly, parameters of dynamic interaction remained significantly different in the MANCOVA (see 3.3) after including RMSSD as a covariate [F(4,136) = 8.2; p<.001].

### First Experiment - Multivariate analysis of co-variance of parameters of heart rate variability controlled for respiration

The MANCOVA for heart rate variability parameters (see 3.2) remained significantly different after including the breathing rate as covariate [F(12,128) = 2.5; p<.005].

### First Experiment - Spearman's rank- order correlation analysis of respiratory data with subscale for positive symptoms of PANSS (P-PANSS)

According to our hypothesis that acute psychotic symptoms influence breathing patterns, we correlated P-PANSS values of patients with respiratory parameters and found a significant positive correlation of positive symptoms with variability indices of respiration: RMSSD_Resp_ (r = 531; p<.009), MF_Resp_ (r = 521; p<.01), RMSSD_TD_ (r = 595; p<.003), LF_TD_ (r = 571; p<.004) and HF_TD_ (r = 584; p<.003). This indicates that higher psychotic scores are associated with higher variability of respiratory parameters.

### First Experiment - Influence of personality traits on autonomic function in relatives and control subjects

A significant difference between groups was found for results of the personality inventory (Freiburger Persönlichkeitsinventar) in the subscale “openness” (p<0.002), indicating that relatives were less open than controls.

Significant correlations between the subscales of the Freiburger Persönlichkeitsinventar and autonomic parameters were observed neither in relatives nor in controls. In particular, cardiovascular measures did not correlate with the subscales for life satisfaction, social orientation, performance orientation, inhibition, excitability, aggression, strain, physical complaints, health sorrows, openness, extroversion and emotionality.

### Second Experiment (stress experiment) - Repeated measures multivariate analysis of variance of respiration and cardiac parameters of the stress experiment

**Figure 4:**
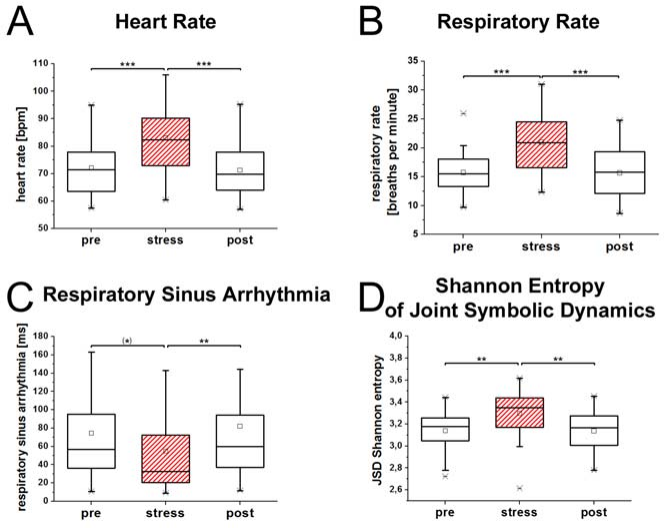
Representative parameters of respiration and heart rate are shown before, during and after a stress task. Healthy controls are characterized by an increased heart rate (A) and respiratory rate during the stress task. Cardiac vagal modulation as shown by RSA (C) is decreased while some evidence was found for increased uncoupling of heart rate and breathing rate during the task (D). Boxes indicate data between the 25th and 75th percentile with the horizontal bar reflecting the median (□ = mean; - = 1st and 99th percentile; x = minimum and maximum of data). Significant differences of Bonferoni corrected pair-wise comparisons are indicated: (*) p = .05; ** p<.01; *** p<.001.

The repeated measures MANOVA for all parameters (mean breathing rate, ratio of inspiration and exhalation, LF_Resp_, MF_Resp_, HF_Resp_, and mean heart rate, RMMSD_HRV_, RSA, Hc_HRV_ as well as JSD Shannon and CCE) showed a significant overall effect for the factor TIME (before, during, and after the stress experiment) [F(8,22) = 8.52; p<.002]. A significant effect of TIME was observed for the following parameters: respiration rate (F = 33; p<.001), ratio of inspiration and exhalation (F = 4.8; p<.02), MF_Resp_ (F = 3.8; p<.04), HF_Resp_ (F = 3.2; p<.05), mean heart rate (F = 28.8; p<.0001), RMMSD_HRV_ (F = 6.4; p<.006), RSA (F = 6.2; p<.005), Hc_HRV_ (F = 9.4; p<.002), and JSD (F = 7.5, p<.002). No difference was observed for LF_Resp_ (p<.23) or CCE (p<.09). Results of Bonferroni corrected pair-wise comparisons between the baseline and stress condition, and between the stress condition and post-stress phases are displayed in Figure 4 and Table 2.

## Discussion

The Greek root of the word “schizophrenia” contains the term ϕρήν (phrēn), referring to “mind”. Interestingly, a second meaning of this word is “diaphragm”, which is closely associated with the breath. In our study, substantial evidence has been presented that breathing rates in acutely psychotic patients are fast, shallow and more variable. In contrast, reduced heart rate variability has been shown both in patients and their healthy first-degree relatives. Moreover, results of our study might suggest that acute induction of stress in healthy subjects leads to fast and shallow breathing as observed in acutely ill patients. Thus, we would like to suggest that alterations in breathing rate and rhythm correspond to the psychotic state, while changes of autonomic cardiac function might be regarded as a trait marker likely to be found in patients and relatives.

We have shown that the regulation of respiration is severely disturbed in patients with schizophrenia. First, patients breathe faster and shallower than controls, as shown previously [16], [18]. The pattern of fast breathing in patients is additionally characterized by a shortening of the exhalation time. This part of the breathing cycle is known to be of special importance for relaxation and vagal modulation [43]. Second, patients ventilate the same amount of air in a certain amount of time, even when breathing is fast and shallow. This is shown in the unchanged minute ventilation volume and the similar oxygenation of hemoglobin, as shown in our study (Table 2). Third, we found that the fast breathing rate is accompanied by an increased amount of middle-, high-, and very high fluctuations in the breathing rhythm. Increased irregularity in breathing rhythms was previously found in patients suffering from panic disorder [44], [45]. However, the pattern seemed completely different to the observed changes in patients suffering from schizophrenia. Fourth, we investigated the depth of breathing, in addition to its rate and rhythm. Shallow breathing is correlated with a smaller tidal volume. However, the variability of tidal volume is not significantly altered, in contrast to the variability of the breathing rate. This might suggest that alterations are mainly reflected in changes of rate and rhythm, and not in the depth of respiration. Fifth, by including RMSSD_HRV_ as a covariate, we have shown that the difference regarding respiratory variability parameters between patients and controls is not dependent on vagal modulation. In accordance with our hypothesis, we found strong positive correlations between changes of breathing patterns and positive symptoms. Many investigators have proposed that limbic/paralimbic circuitry underlie the changes in breathing patterns that accompany cognitive tasks and emotional states in healthy subjects [12], [46], [47]. It is highly likely that a dysregulation of arousal, as suggested in paranoid schizophrenia in amygdalae-prefrontal circuits, might contribute to the correlation of psychopathology and breathing alterations [8]. This might additionally explain why alterations in breathing rate and rhythm were observed in patients only, and not in healthy relatives.

Breathing is a core physiological rhythm which is able to influence peripheral physiological processes such as heart rate, blood pressure, oxygenation of hemoglobin, metabolism and central states of arousal. We suggest that future research needs to investigate the sequelae of altered breathing rates for patients on the one hand and for signal detection in other research areas on the other. For instance, a recent study by Rachow et al. [22] on heart rate and electrodermal activity in patients with schizophrenia emphasized the enormous influence of breathing rates. In this study, group differences in electrodermal activity between patients and controls were strongly dependent on the breathing rates of the patients. In addition, it has been shown that even subtle changes in breathing depth and rate, which occur naturally during rest at very low temporal frequencies [48], can lead to significant signal changes in functional imaging [49]. Thus, future research needs to include breathing rate as a potential confounding factor. The clinical significance of increased variability of respiration for patients with schizophrenia also needs to be investigated in the future. First of all, more research is warranted to investigate whether changes of breathing are restricted to the acute psychotic state or whether they might persist over time. Secondly, respiration has been used in many biofeedback studies to increase heart rate variability. Therefore, future studies need to follow this line of evidence to elucidate whether breathing might be a therapeutic option for influencing reduced HRV in patients.

Overwhelming evidence of reduced HRV in patients with schizophrenia has accumulated [9], [50], [51], [52], [53], [54], [55], [56], [57]. This was described in the acute [9], [50], [58], [59], [60], [61] and chronic states [57], [62]. Moreover, autonomic dysfunction has been reported in healthy first-degree relatives as well [4], [5], [6], [11]. Our study extends previous investigations by demonstrating that HRV is reduced in patients and their relatives when compared to controls. This seems to be not dependent on the rate of respiration as shown by the transfer function of RSA (Figure 2F) or the statistical analysis including respiration rate as a covariate of HRV parameters. This analysis indicates that reduced HRV in patients with schizophrenia is not caused by increased rates of respiration. We conclude that a genetic background might be assumed for the pattern of HRV parameters observed in relatives and patients, but not for the respiratory component.

Respiratory neurons within the brainstem receive regulating synaptic input from non-respiratory regions such as the motor cortex, pontine and medullary reticular formations, cerebellum, hypothalamus, limbic/paralimbic regions, and cardiovascular regions of the brainstem. These non-respiratory modulatory inputs adapt breathing rhythm and pattern to effective cardio-respiratory interactions [63]. We exploited two non-linear measures to investigate the interdependency of cardio-respiratory interactions (coupling, complexity) of breathing and heart rate time series in all three groups. We observed an impaired coupling, revealed by increased uncoupling function and a complexity of cardio-respiratory interactions in patients. We propose that decreased vagal activity within the brainstem or its suppression from higher regulatory centers might account for this finding [8]. Again, descending projections from the central nucleus of the amygdaloid complex (AC) or other limbic/paralimbic regions might be responsible [64]. In addition, significant endogenous dopaminergic modulation of breathing is putatively altered in acute psychosis [63].

We suggest here that acute internal stress leads to observed changes of breathing and heart rate. Therefore, we included an additional experiment with controls and subjected them to a stress task known to influence HRV parameters [42]. We observed that breathing and HRV parameters shifted drastically in a similar direction to that found in patients suffering from schizophrenia. Thus, heart and breathing rate increased, variability of heart rate decreased and coupling of breathing and heart rate time series was reduced during stress. In addition, the duration of the exhalation time was reduced, as seen in patients. Our experiment illustrates therefore that some aspects of autonomic dysregulation are due to a high level of arousal [8]. However, some discrepancies between respiration patterns of patients and controls were observed. In contrast to patients, we found no increase in variability of breathing patterns during the stress task. This might be suggestive of additional regulatory mechanisms influencing the autonomic patterns in patients. Future studies need to investigate whether differences are due to chronic arousal in patients or whether other physiological mechanisms might be involved. Functional imaging should be applied to study neuronal underpinnings of breathing in healthy controls during stress and in patients with schizophrenia to reveal the physiological basis of observed findings.

Some limitations need to be addressed. Patients were investigated in the acute state and we can only speculate on patterns of regulation after recovery or remission of symptoms. Furthermore, although an attempt was made to match control subjects to patients, it is very difficult to find healthy heavy smokers in this age group. In addition, the information on the amount of alcohol consumption and smoking was obtained by means of questionnaires only. Future studies need to exploit more objective measures, such as CO-binding to hemoglobin. Our patients were relatively young and were investigated for respiratory diseases to exclude potential confounding factors due to smoking. In contrast to alterations seen in our patients, smoking leads to a prolongation of exhalation times and does not explain our results [65]. However, a group of non-smoking patients with schizophrenia should be investigated to rule out any potential confounding influence. In addition, our study only assessed blood oxygen saturation levels. We did not assess how pCO_2_, pH, or other factors influence ventilation in patients with schizophrenia. This needs to be analyzed in detail in future studies to exclude other potential sources of altered breathing patterns.

In conclusion, we report increased variability in the regulation of respiration and decreased cardio-respiratory coupling in patients with schizophrenia in comparison to controls. We suggest that this finding is of importance for physiological regulation in patients, and we assume that many biological signals are influenced by altered breathing patterns and rates. In contrast to comparable HRV changes in patients and healthy first-degree relatives described here, changes of respiration were observed in patients only, and might reflect arousal in acutely ill patients. This assumption is supported by observed changes of breathing and cardiac regulation in healthy subjects during stress tasks.
